# Comparison of random forest methods for conditional average treatment effect estimation with a continuous treatment

**DOI:** 10.1177/09622802241275401

**Published:** 2024-10-09

**Authors:** Sami Tabib, Denis Larocque

**Affiliations:** Department of Decision Sciences, 10014 HEC Montréal, Montréal, Canada

**Keywords:** Conditional average treatment effect (CATE), continuous treatment, random forest, tree-based method, confounding effect, colliding effect, local centering, uplift modeling, incremental modeling, causal modeling, ensemble method

## Abstract

We are addressing the problem of estimating conditional average treatment effects with a continuous treatment and a continuous response, using random forests. We explore two general approaches: building trees with a split rule that seeks to increase the heterogeneity of the treatment effect estimation and building trees to predict Y as a proxy target variable. We conduct a simulation study to investigate several aspects including the presence or absence of confounding and colliding effects and the merits of locally centering the treatment and/or the response. Our study incorporates both existing and new implementations of random forests. The results indicate that locally centering both the response and treatment variables is generally the best strategy, and both general approaches are viable. Additionally, we provide an illustration using data from the 1987 National Medical Expenditure Survey.

## 1. Introduction

### 1.1. Background

This article investigates the effect of a treatment in a population, where treatment is considered in a broad sense (e.g. a specific drug, a marketing action, and an exposure to a risk factor). Large-scale decisions (e.g. those made by public health agencies) often rely on population-averaged effects. However, the effect of the treatment is frequently heterogeneous in the population. In such cases, individual-level decisions should account for the specific characteristics of each subject. Estimating the conditional average treatment effect (CATE) is a highly active area of research.^1^ This problem is typically studied under the causal modeling framework.^2^ The same problem appears in the business literature under the name “uplift” or “incremental” modeling.^3^

Tree-based methods are especially well-adapted to the problem of CATE estimation because they can be designed to adaptively find subgroups with similar treatment effects within the population. While the vast majority of the literature focuses on binary treatment variables, it is important to note that continuous treatments (such as doses of a drug or levels of exposure to risk factors) are commonly encountered in practice.^4^ However, limited research exists on CATE estimation using tree-based methods specifically for continuous treatments.^5–7^

### 1.2. Motivation and related work

One important challenge is that the target of interest, the CATE, is unknown even in the observed sample. Consequently, it is not possible to construct trees using a split rule that directly measures the prediction error for the CATE. The following two approaches are possible to overcome this challenge:

- Approach 1. Build trees with a split rule that seeks to increase the heterogeneity of the CATE estimation.
- Approach 2. Build trees to predict a proxy target variable, usually the response Y .

Approach 1 stems from the modern view of the random forest (RF) operating mechanism, which sees it as a “weight-generating machine” trained to find a set of observations “close” to the one for which an estimation (or prediction) is needed. These weights are commonly referred to as nearest-neighbor forest weights (NNFWs).^8^ A related concept is the “bag of observations for prediction” (BOP).^9^ At a high level, the general modern framework to develop new forest-type methods involves the following steps: (a) design a specialized split rule tailored to the problem at hand; (b) build a forest using this specialized split rule; (c) for a new observation, obtain the NNFWs (or the BOP) and compute the desired parameter using them. Generalized RFs (GRFs)^5^ is a method that follows this framework. The same article provides a theoretical justification for using a split criterion that seeks to increase the heterogeneity in the parameter of interest. This approach can be seen as an approximation of a split rule that aims to minimize the least-squares criterion for this parameter (refer to Proposition 1 in that article). In practice, this approach performs well and has been applied in various contexts. For instance, an early example involves using the log-rank test as the split rule with survival data.^10^ The log-rank test aims to maximize the heterogeneity of the conditional survival function (an unknown quantity for all subjects). Other examples of this approach in different settings include works by Moradian et al.,^11^ Tabib and Larocque,^9^ and Alakuş et al.^12,13^

It is also possible to use Y as the target variable to build the trees and then utilize the NNFWs to compute the estimation of the main target quantity, specifically the CATE. This corresponds to Approach 2 mentioned earlier. An early example of this approach is the quantile regression forest (QRF) proposed by Meinshausen and Ridgeway.^14^ In the context of QRF, the main targets are conditional quantiles of the response variable Y , which remain unknown for all observations. QRF employs a regression forest with Y as the target to find the NNFWs, which are then used to estimate the conditional quantiles. The reason why this approach is reasonable is that the link between the covariates and the proxy target and the link between the covariates and the true target can be sufficiently similar so that the forest with the proxy target produces good sets of local observations for the final estimation. Interestingly, even using random neighborhoods in the covariate’s space, such as extremely randomized trees,^15^ can perform well.

Local centering of the response and/or the treatment variable is now recognized as being an important strategy for improving performance, especially in the presence of confounding,^16^ which is one of the main challenges for CATE estimation. However, less attention has been given to collider bias^17,18^ even though including a collider variable in the model can have a detrimental effect.

Motivated by the above discussion, this article presents an empirical study to investigate RFs for CATE estimation. The study incorporates the following elements and characteristics: (a) it utilizes a continuous treatment, as this situation has been less studied; (b) it includes RFs from both approaches described earlier; (c) it employs data-generating processes (DGPs) that incorporate confounding and colliding effects; (d) it explores different variants for preprocessing the data by locally centering the treatment and response.

The work most closely related to this article is Dandl et al.^16^ They also investigate several variants of RFs to estimate CATE, but in the case of a binary treatment. In our study, we focus on a continuous treatment, investigate additional aspects such as collider effects, and consider other variants of RF, including new implementations. This allows for more direct comparisons between the methods. A discussion comparing the findings of Dandl et al.^16^ and ours is given in Section 3.2.1.

The article is organized as follows. Section 2 describes the problem and methods. Section 3 presents the results from the simulation study. Section 4 provides an illustration using real data. Finally, a discussion and conclusion are presented in Section 5.

## 2. Methods

In this section, we define the problem and notation, and describe the investigated methods.

### 2.1. Notation, data, and CATE definition

Let us consider a sample of N observations denoted as (Y,X,G) , where Y is a continuous response, $X=(X_{1},\ldots ,X_{p})$ is a vector of p covariates, and G is a treatment variable. Initially, let us assume that the treatment is binary, taking values G=0 (non-treated) or G=1 (treated), which corresponds to the classical problem. In this case, the usual definition of the CATE is given by:

(1) E[Y|X=x,G=1]−E[Y|X=x,G=0]

However, in this paper, we assume that G is a continuous treatment variable. We assume the following model relating the response to the treatment and covariates:

(2) E[Y|X=x,G=g]=μ(x)+τ(x)g

Hence, Y is a linear function of G but the coefficients can vary with X . Thus, it is a varying coefficients model. For a given X=x , we define the CATE as τ(x) . Specifically, τ(x) represents the mean increase in the response for a one-unit increase in the treatment for a subject with covariates x . This definition reduces to (1) when the treatment is binary. Note that it is also possible to assume a fully parametric model by specifying, for example,

$$\begin{array}{r} \mu (x)=\alpha_{0}+\alpha_{1}x_{1}+\cdots +\alpha_{p}x_{p} \end{array}$$

and

$$\begin{array}{r} \tau (x)=\gamma_{0}+\gamma_{1}x_{1}+\cdots +\gamma_{p}x_{p} \end{array}$$

This model corresponds to a simple linear model containing all main effects and all interactions between the treatment and the covariates. However, specifying the right parametric model is usually difficult, especially when several covariates are available. This is why we focus on RFs, which avoid explicit specifications of μ(x) and τ(x) . From this point of view, model (2) is semi-parametric.

Still, assuming a linear model in G , even with varying coefficients, may appear too restrictive in some situations. Fortunately, the methodology studied in this article can be generalized to allow more flexible models, and this is discussed in Section 5.

The data-generating processes we use satisfy the usual assumptions of positivity and unconfoundedness. The first one states that for any given covariates, all treatment values are possible, that is, $f_{G|X}(g|x)>0$ , for all g , where $f_{G|X}$ is the conditional density of G given X . The second assumption states that all confounders are included in X , meaning that, conditional on the covariates, the treatment value is independent of the potential outcomes (one for each possible value of the treatment). In fact, these assumptions are required to derive CATE definitions such as (1) and (2) from a potential outcome framework.

### 2.2. Description of the RF methods

To estimate the CATE at X=x , that is, τ(x) , we would ideally have a large sample of subjects with the same covariate pattern X=x . In that case, we could directly fit the model

(3) Y=μ+τG+ϵ

with that sample and directly obtain an estimate of the CATE. However, such a sample is often not available, especially in observational data where many covariates are included. One approach is to use a local model by considering a sample of observations with covariate values in the neighborhood of x . This is where RFs come into play, as they are powerful methods for identifying such neighborhoods. In fact, as discussed in the Introduction, the modern view considers RF as a weight-generating machine that identifies locally similar observations.

Given a training sample (Y,X,G) , assume we have built an RF of B trees, and we want to estimate the CATE at a new point $x_{\mathrm{new}}$ , that is, $\tau (x_{\mathrm{new}})$ . Following Tabib and Larocque,^9^ let $S_{b}(x_{\mathrm{new}})$ be the training observations that fall into the same terminal node as $x_{\mathrm{new}}$ for the bth tree. Note that any given observation can be present multiple times in $S_{b}(x_{\mathrm{new}})$ when a bootstrap sample is used to build the tree. The set of all these observations for the RF is called the BOP, formally defined as

$$\begin{array}{r} \text{BOP}(x_{\mathrm{new}})=\overset{B}{\underset{b=1}{⋃}}S_{b}(x_{\mathrm{new}}) \end{array}$$

This BOP represents a collection of locally similar observations that we use to estimate any desired quantity, including $\tau (x_{\mathrm{new}})$ . Specifically, we fit model (3) using $\text{BOP}(x_{\mathrm{new}})$ as the sample. The obtained $\hat{\tau }$ is the estimated $\hat{\tau }(x_{\mathrm{new}})$ .

Given this general approach, the main question is how to build the RF? We explore variants of the two approaches presented in the Introduction: Approach 1 and Approach 2. While existing implementations are available, they rely on different tree-building algorithms, which can introduce confounding factors when comparing methods. To ensure a more direct and fair comparison, we have implemented our own variants, all based on the same tree-building algorithm following the classification and regression trees (CART) paradigm proposed by Breiman et al.^19^

#### 2.2.1. Methods based on the same tree-building architecture

In our study, we implemented two different methods for building decision trees to estimate the CATE. Here is a description of these methods. At a given node t , let $t_{L}$ ( $t_{R}$ ) be the set of indices of the observations that are in the left (right) node for a candidate split, and let $N_{L}$ ( $N_{R}$ ) be the size (i.e. number of observations) of the left (right) node.

With Approach 1, we want to build trees that seek to increase the heterogeneity of the CATE estimation. To achieve this, we use the following split rule:

(4) $$\sqrt{N_{R}N_{L}}|{\hat{\tau }}_{L}-{\hat{\tau }}_{R}|$$

where ${\hat{\tau }}_{L}$ ( ${\hat{\tau }}_{R}$ ) is the estimated τ in model (3) fitted with the observations in the left (right) node. The best split is the one maximizing (4). This method is called HET.

With Approach 2, we use Y as the proxy target variable. However, we also exploit the assumption (2) that the response is linearly related to the treatment given the covariates, and use the split rule

(5) $$\sum_{i\in t_{L}}(Y_{i}-{\hat{\mu }}_{L}-{\hat{\tau }}_{L}G_{i}{)}^{2}+\sum_{i\in t_{R}}(Y_{i}-{\hat{\mu }}_{R}-{\hat{\tau }}_{R}G_{i}{)}^{2}$$

where ${\hat{\mu }}_{L}$ and ${\hat{\tau }}_{L}$ are the estimated parameters of model (3) for the left node observations ( ${\hat{\mu }}_{R}$ and ${\hat{\tau }}_{R}$ are defined similarly for the right node). This type of method is known as “MOdel Based recursive partitioning” or MOB and was introduced by Zeileis et al.^20^ The original MOB method is based on the “conditional inference trees” paradigm and is implemented in the packages party and partykit.^21^ The variant described above can be seen as a CART version of MOB and will be called CMB.

The two methods described above were implemented using the RandomForestSRC package,^22^ leveraging its custom split rule feature. These split rules were specifically developed in C++. To do it, we have created a new “Incremental” method family which is characterized by the fact that the response is given by a pair of variables, the response ( Y ) and the continuous treatment ( G ). We used OpenMP (OpenMP Architecture Review Board^23^) to allow parallel treatment to optimize overall performances. Once the forest is built, we use a C++ code to extract the BOP and calculate the estimated treatment effect.

#### 2.2.2. Methods based on existing packages

For Approach 1, the grf package has a function to fit an RF with a continuous treatment and a continuous response. The underlying idea of the GRF method is to use split rules like (4). However, the main difference is that grf employs a faster but approximate split rule based on the so-called “gradient tree algorithm.” Another difference is that grf uses by default the concept of “honesty” that builds trees on subsamples of the training data. In our simulations, we directly use the causal_forest function from the grf package. However, for a fair comparison with other methods, we set honesty = FALSE. Leaving honesty = TRUE tends to result in worse performance in the scenarios considered. Detailed results comparing both versions are presented in the supplemental material. Once the forest is built, we can directly use the predict function to obtain estimated treatment effects for new observations. Note that Wager and Athey^24^ proposed a related method for a binary treatment that uses directly a split rule like (4), without local centering.

Another method included in our study is a forest of MOB trees built using the partykit package. The target is Y and (3) is used as the within-node model. The partykit package builds a single tree, not a forest. We build a forest using the standard forest algorithm, that is, we build independent MOB trees using bootstrap samples. Once the MOB forest is built, for a new observation, we extract the BOP and calculate the treatment effect as described above. The MOB split rule involves two stages. The first stage seeks the covariate that has the most instability with respect to both μ and τ . This step is akin to selecting the covariate that maximizes a joint heterogeneity measure, aligning with Approach 1. In the second stage, the best split with this variable is found as the one that minimizes the least-squares criterion to predict Y , aligning with Approach 2. From this perspective, this is a hybrid method combining elements from both Approaches 1 and 2. In contrast, the CMB method presented above also employs a linear within-node model for the treatment effect but aims at minimizing directly the least-squares criterion to predict Y . For further discussions on these aspects, refer to Seibold et al.,^25,26^ and Dandl et al.^16^

#### 2.2.3. Local centering variants

As highlighted in the introduction, locally centering the response and the treatment variables is often crucial for enhancing performance. Let ${\tilde{Y}}_{c}$ be the centered version of Y obtained as ${\tilde{Y}}_{c}=Y-\hat{m}(x),$ where $\hat{m}(x)$ is an estimation of the conditional mean of the response m(x)=E[Y|X=x] . Likewise, let ${\tilde{G}}_{c}$ be the centered version of G obtained as ${\tilde{G}}_{c}=G-\hat{\pi }(x),$ where $\hat{\pi }(x)$ is an estimation of the conditional mean of the treatment π(x)=E[G|X=x] , also known as the propensity score. We use RFs from the package randomForestSRC to estimate m(x) and π(x) for the HET, CMB, and MOB methods. The out-of-bag predictions are used for centering. This is a preprocessing step performed right at the beginning. Subsequently, the centered versions ${\tilde{Y}}_{c}$ and ${\tilde{G}}_{c}$ are used to build the RFs to estimate the CATE. All combinations are considered in our study. That is, (i) Y and G are used (no centering), (ii) ${\tilde{Y}}_{c}$ and G are used (only the response is centered), (iii) Y and ${\tilde{G}}_{c}$ are used (only the treatment is centered), and (iv) ${\tilde{Y}}_{c}$ and ${\tilde{G}}_{c}$ are used (both are centered). Notably, the GRF method already centers Y and G using RFs by default. Consequently, only this variant is considered in our study.

#### 2.2.4. Summary of the methods and specific details for the RF parameters

Table 1 summarizes the methods considered in the study. For methods, HET, CMB, and MOB, four variants are considered by having Y and G locally centered or not. For GRF, only the default method with Y and G centered is considered. Consequently, we have 13 methods in total ( 3×4+1 ).

**Table 1.** Methods investigated in the simulation study.

	Approach 1		Approach 2
	Max heterogeneity	Hybrid	Proxy target
Comparable implementations	HET (split rule (4))		CMB (split rule (5))
Existing packages	GRF (package grf)	MOB (package partykit)	

For all forests, the number of variables selected at each node (i.e. mtry) is set to 5. The size of the trees is controlled by nodesize in randomForestSRC, min.node.size in grf, and minsize in partykit. These values are all set to 30. As mentioned above, we set honesty = FALSE for the GRF method. The number of trees in a forest is 100. The other parameters are left to their default values.

## 3. Simulation study

In this section, we present the results of a simulation study designed to evaluate the relative performance of the methods to estimate the CATE introduced in the preceding section. We first describe the simulation design and then present the results.

### 3.1. Simulation design

The data-generating processes (DGPs) employed in our study serve the purpose of investigating confounding and colliding effects. Additionally, we incorporate various functional forms for the treatment effect. There are five covariates $X_{1},\ldots ,X_{5}$ . The first four are independent and uniformly distributed in the interval [0,10] .

The response Y is generated according to

$$\begin{array}{r} Y=h(G,X_{1},\ldots ,X_{4})+\epsilon \end{array}$$

where ϵ s are independent random errors from a normal distribution with mean 0 and standard deviation 0.5. The function h is given by

$$\begin{array}{r} h(G,X_{1},\ldots ,X_{4})=\left\{ \begin{array}{l} 1+\tau (X_{4})\times G\;\text{if}\;X_{1}\leq 5\;\&X_{3}\;\leq \;5\\ 2+\tau (X_{4})\times G\;\text{if}\;X_{1}\leq 5\;\&X_{3}\;>\;5\\ 3+\tau (X_{4})\times G\;\text{if}\;X_{1}>5\;\&X_{2}\;\leq \;5\\ 4+\tau (X_{4})\times G\;\text{if}\;X_{1}>5\;\&X_{2}\;>\;5 \end{array}\right. \end{array}$$

Three different types of treatment effects are used, each one depending on $X_{4}$ . They are

1. Step treatment effect
$$\begin{array}{r} \tau (X_{4})=\left\{ \begin{array}{ll} 1 & \;\text{if}\;X_{4}\;<\;5\\ 5 & \;\text{if}\;X_{4}\;\geq \;5 \end{array}\right. \end{array}$$
2. Linear treatment effect
$$\begin{array}{r} \tau (X_{4})=X_{4}\;/\;2 \end{array}$$
3. Quadratic treatment effect
$$\begin{array}{r} \tau (X_{4})=(X_{4}-5{)}^{2}\;/\;5 \end{array}$$

Two distributions for the treatment variable G are used.

1. Randomly uniform. In this case, G is uniformly distributed between 0 and 1. Instances where G is generated in this manner correspond to scenarios with no confounding effects.
2. Correlated with the covariate $X_{1}$ (confounder). In this case, $G=0.6-X_{1}/50+\epsilon_{G},$ where $\epsilon_{G}$ follows a normal distribution with a mean of 0 and a standard deviation of 0.17 (weak confounding) or 0.07 (strong confounding). Values outside [0,1] are truncated at the boundaries of this interval. In these scenarios, $X_{1}$ is a confounder.

The fifth covariate $X_{5}$ is generated according to one of two distributions.

1. Independent. In this case, $X_{5}$ is uniformly distributed between 0 and 10 and is not linked to the other covariates and Y . Instances where $X_{5}$ is generated in this manner correspond to scenarios with no colliding effects.
2. Collider. In this case, $X_{5}=h(G,X_{1},\ldots ,X_{4})+\epsilon_{X},$ where $\epsilon_{X}$ follows a normal distribution with a mean of 0 and a standard deviation of 4 (weak collider) or 1.5 (strong collider). In these scenarios, $X_{5}$ is a collider.

In total, we have 27 scenarios (three types of treatment effects (step, linear, and quadratic) × 3 confounding effects (none, weak, and strong) × 3 collider effects (none, weak, and strong)). The terms “weak” and “strong” are inherently subjective and serve as convenient discussion aids. To provide clarity, let us establish some reference points. In typical samples with a weak confounding effect, $\text{cor}(X_{1},G)$ varies between − 0.31 and − 0.33. In typical samples with a strong confounding effect, $\text{cor}(X_{1},G)$ varies between − 0.63 and − 0.64. In typical samples with a weak colliding effect, $\text{cor}(X_{5},Y)$ varies between 0.28 and 0.39, depending on the type of treatment effect. In typical samples with a strong colliding effect, $\text{cor}(X_{5},Y)$ varies between 0.6 and 0.75, depending on the type of treatment effect.

The number of repetitions for each scenario is 100. The training data sample size is $n_{\mathrm{train}}=1000$ . For a given RFin a given repetition, the performance is evaluated with an independent test set of size $n_{test}=500$ with

$$\begin{array}{r} {\text{MSE}}_{\mathrm{CATE}}=\frac{1}{n_{\mathrm{test}}}\sum_{i=1}^{n_{\mathrm{test}}}({\hat{\tau }}_{i}-\tau_{i}{)}^{2} \end{array}$$

where ${\hat{\tau }}_{i}$ and $\tau_{i}$ are the estimated and true treatment effects. Smaller values of ${\text{MSE}}_{\mathrm{CATE}}$ indicate a better performance.

### 3.2. Simulation results

While the main article contains figures that sufficiently convey the primary findings of this study, we also provide additional figures in a separate supplemental material document, including discussions based on the mean absolute error and the C-index as alternative performance measures, as well as comments about the Monte–Carlo error. To ease the discussion, we will denote by NoC, Cy, Cg, and Cyg, the no centering, center Y (response) only, center G (treatment) only, and center both Y and G variants. Let us begin by summarizing the key general findings.

1. Locally centering both the response and treatment (Cyg) is generally preferable for all methods considered.
2. When considering the Cyg variants, both approaches, namely splitting based on maximizing the heterogeneity of the treatment effect and splitting to predict the response, are viable. Neither approach dominates the other across all scenarios.
3. In the scenarios considered, the presence of a confounder has a more detrimental impact on performance than that of a collider.

**Figure 1.**
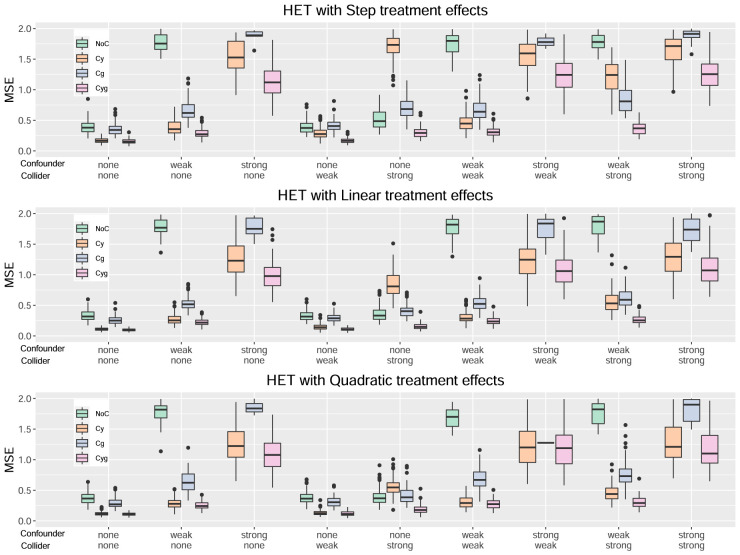
Results for the method HET.

**Figure 2.**
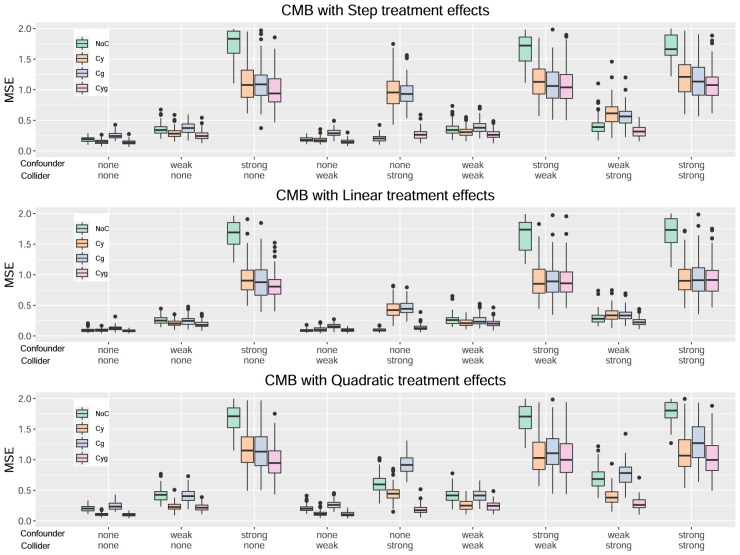
Results for the method CMB.

**Figure 3.**
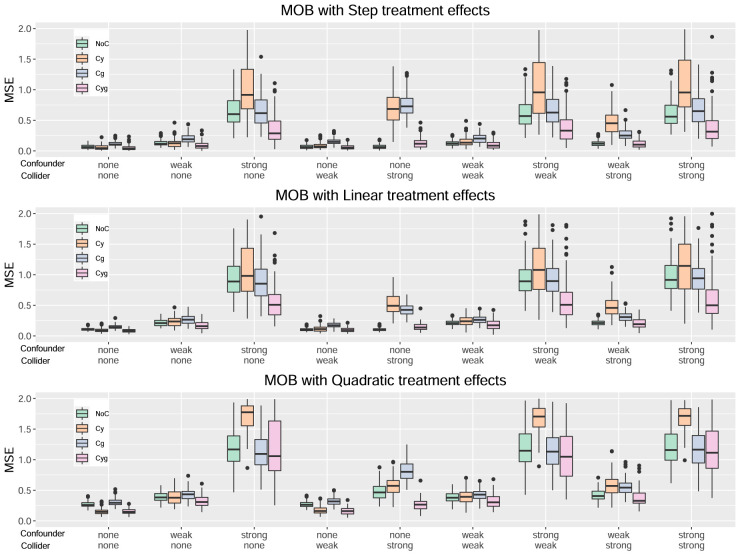
Results for the method MOB.

**Figure 4.**
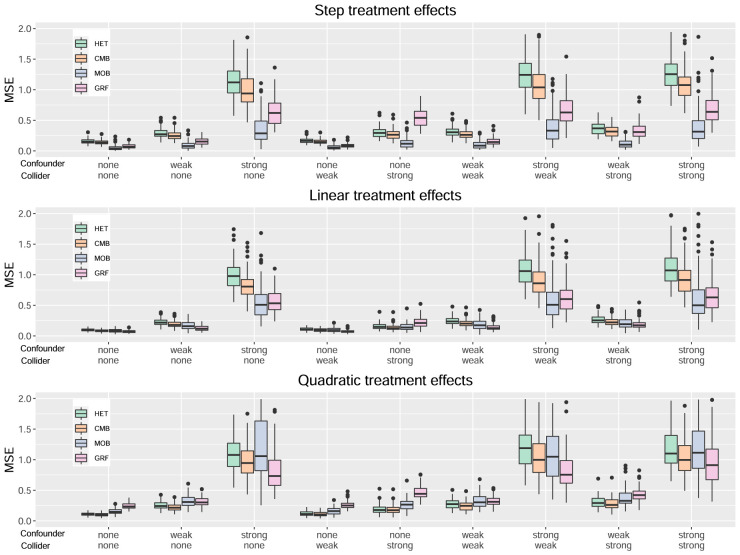
Results for all methods, Cyg variant for HET, CMB, and MOB.

Figures 1 to 4 serve to visualize these general findings and to provide more specific insights. The box plots show the distribution of the mean squared error (MSE) over the 100 simulation runs. Figures 1 to 3 show the performance of all centering variants for a specific method (HET, CMB, and MOB). Recall that only the Cyg variant is considered for GRF since this is the default for this method. Figure 4 compares the four methods HET, CMB, MOB, and GRF, but only their Cyg variant. To aid interpretation, we truncate the y -axis at 2. Hence, some of the box plots are only partly shown and some are even not apparent. In the latter case, it means that they are above 2. For untruncated versions of these figures, please refer to the supplemental material.

For the HET method, from Figure 1, we can observe that the Cyg variant generally performs the best (based on the median mean squared error (MSE)). We can observe that a strong confounder has a more adverse effect on NoC. A strong collider has a more adverse effect on Cy and Cg. Interestingly, the two cases where NoC is slightly better than Cyg are the step and linear treatment effects with a strong collider and no confounder. Another finding, that is also true for the other methods, is that the confounder and collider effects do not interact in a way to multiply their negative effects. Their combined effect is at most additive or even less than that in some cases.

From Figure 2, the patterns observed for the CMB method are very similar to the ones for HET, leading to the same conclusions.

For the MOB method, from Figure 3, the Cyg variant once again emerges as the best choice. We can again observe that a strong collider has a more adverse effect on Cy and Cg. However, unlike the two previous methods, NoC is not the most affected by a strong confounder, as it is Cy this time.

Formal comparisons based on paired-sample t -tests are presented in the supplemental material, with the same conclusion that the Cyg variant is the best one for all methods.

In Figure 4, we directly compare the Cyg variant (the best one) across the four methods. To gain a comprehensive perspective, Table 2 provides the number of times each method has a given rank when we rank the four methods separately for each of the 27 scenarios. A rank of 1 indicates the best performance. Globally, MOB emerges as the top performer in 12 out of 27 scenarios, GRF in eight scenarios, and CMB in seven scenarios. MOB has also the lowest average rank of 2.07, followed by CMB at 2.22 and closely by GRF at 2.3. But keep in mind that the performances are very close in several scenarios as seen in Figure 4. For example, even if HET is not the single best method in any scenario, its performance is very close to the one of CMB in general and, consequently, it often comes in second place in scenarios where CMB is the best method (see Table 3 for more details).

Looking at each type of treatment effect separately, we see that MOB has the best performance for the step treatment effect (upper plot). For the linear treatment effect (middle plot), the best method is either MOB or GRF. For the quadratic treatment effect (lower plot), the best method is either CMB or GRF. We also note that GRF is the worst when there is a strong collider alone. But on the other hand, it is one of the two best methods when there is a strong confounder alone.

Comparing two methods at a time is also interesting. Table 3 presents the results for interesting pairs which are discussed in the following.

The two methods that are the more directly comparable without interference from specific aspects of the tree and forest implementations are HET and CMB. Recall that these two methods share exactly the same tree and forest building architecture, and only the splitting rules they use are different. We can see in Figure 4 that CMB and HET are very close but CMB has a slight advantage in all scenarios and most notably in the ones with a strong confounder. The paired-sample t -tests indicate that the mean MSEs are significantly different across all 27 scenarios. However, the magnitude of the difference in mean MSE is consistently <20% in each case.

The direct comparison of MOB and CMB is also of interest. MOB is a hybrid approach between Approaches 1 and 2. CMB is a “pure” Approach 2 method. MOB significantly outperforms CMB in 15 out of 27 scenarios and the difference in mean MSE exceeds 20% for 12 of them. Conversely, CMB significantly outperforms MOB in 10 out of 27 scenarios, and the difference in mean MSE exceeds 20% for 7 of them. MOB is generally better than CMB for the step treatment effects scenarios but the opposite occurs for the quadratic treatment effect. This highlights that even without incorporating directly the heterogeneity maximizing component, a method based on a proxy target can be competitive.

The direct comparison between MOB and GRF is also of interest as they are two existing methods prior to this article. In 14 out of 27 scenarios, MOB significantly outperforms the CMB. Conversely, in eight out of 27 scenarios, CMB significantly outperforms MOB. Further discussions related to Dandl et al.^16^ can be found in the next subsection.

The direct comparison between GRF and HET is also of interest as they are two methods from Approach 1. They are based on very similar ideas and rely on the CART paradigm. HET uses directly a split rule that seeks to maximize the heterogeneity in the treatment effect, while GRF uses an approximate version of it. Other specific details in the GRF implementation might explain the differences we find but, in the end, GRF significantly outperforms HET in 19 out of 27 scenarios. Conversely, HET significantly outperforms GRF in eight scenarios.

#### 3.2.1. Comparing our results to the ones in Dandl et al.^16^

In their empirical investigation, Dandl et al.^16^ explore various variants of RFs for estimating treatment effects in the context of binary treatments. In our study, we extend this analysis by focusing on continuous treatments and examining additional aspects, including collider effects. We also include other variants such as the CART version of MOB. It is possible to compare some of the general conclusions of both articles. The variant Cyg of MOB we use in our study is basically the method called $\text{mob}(\hat{W},\hat{Y})$ in Dandl et al.^16^ Also, what we refer to as GRF in this article is called cf in Dandl et al.^16^ In Dandl et al.,^16^ $\text{mob}(\hat{W},\hat{Y})$ generally outperforms the no centering (NoC) and the center the treatment only (Cg) variants. We have the same general conclusion not only for MOB, but also for the other methods considered (HET and CMB). Moreover, we also find that the Cy variant can be better than the Cg variant in some scenarios. But ultimately, Cyg is the best variant. In Dandl et al.,^16^ $\text{mob}(\hat{W},\hat{Y})$ outperforms cf in three of their four setups, or more precisely, in 12 out of their 16 scenarios. We have a similar conclusion as MOB (Cyg variant) is better than GRF in 18 out of the 27 scenarios we considered.

## 4. Real data example

In this section, we present an illustration using data from the 1987 National Medical Expenditure Survey. The goal is to investigate the impact of smoking on medical expenditure. These data have been analyzed in Johnson et al.,^27^ Imai and Van Dyk,^28^ and Hahn et al.^29^ We use the data available in the package causaldrf.^30^

**Table 2.** Overall summary: Number of times each method has a given rank when we rank the four methods, with respect to the median mean squared error (MSE), for each of the 27 scenarios. Lower is better.

	Method			
Rank	HET	CMB	MOB	GRF
1	0	7	12	8
2	6	7	4	10
3	4	13	8	2
4	17	0	3	7
Mean rank	3.41	2.22	2.07	2.30

**Table 3.** Some relevant paired comparisons.

	Mean MSE is significantly different and the difference (in %) is		
Pair	>20%	[10%, 20%]	[0, 10%]
CMB better than HET	0	23	4
HET better than CMB	0	0	0
MOB better than CMB	12	1	2
CMB better than MOB	7	1	2
MOB better than GRF	13	1	0
GRF better than MOB	6	1	1
GRF better than HET	17	2	0
HET better than GRF	7	1	0

MSE: mean squared error.

*Note*: Paired-sample t -tests comparing the mean MSE over the 100 repetitions are conducted at the 5% significance level. The results indicate the number of times one method significantly outperforms the other, along with the corresponding percentage difference in mean MSE, across all 27 scenarios.

### 4.1. Presentation of the covariates, treatment variable, and outcome

For this example, we use the following covariates and keep the names provided in the package to facilitate the description and references:

- AGESMOKE: age when the individual started smoking (in years).
- LASTAGE: age in years at the time of the survey (in years).
- MALE: gender (male or female).
- RACE3: black, white or other.
- BELTUSE: use a seat belt when in a car regularly (yes or no).
- EDUCATE education level (college graduate, some college, high school graduate, and other).
- MARITAL: marital status (married, widowed, divorced, separated, and never married).
- POVSTALB: poverty status (poor, near poor, low income, middle income, and high income).

The observed response is the annual medical expenditure, but this variable exhibits significant skewness. Consequently, following the approach of Imai and Van Dyk^28^ and Hahn et al.,^29^ we take its natural logarithm as the outcome. The level of smoking serves as our treatment variable. As proposed in Johnson et al.,^27^ we capture the smoking effect using the variable “packyears,” which represents the number of cigarettes per day times the number of years smoked divided by 20. Finally, we made a standardization to the packyears variable to ensure it is in the [0,1] range. Following Johnson et al.^27^ and Imai and Van Dyk,^28^ we retain only patients with positive medical expenditures and exclude records with missing values. Our final sample consists of n=8263 subjects.

### 4.2. Data analysis

We employ the same four methods used in the simulation study to estimate treatment effects. For HET, CMB, and MOB, the Cyg (center the response and the treatment) variant is used. However, for reasons explained below, we also consider the NoC variant for MOB and the GRF variant with honesty = TRUE. A 10-fold cross-validation scheme is used. In the end, we obtain one out-of-sample estimated treatment effect per subject and per method.

**Figure 5.**
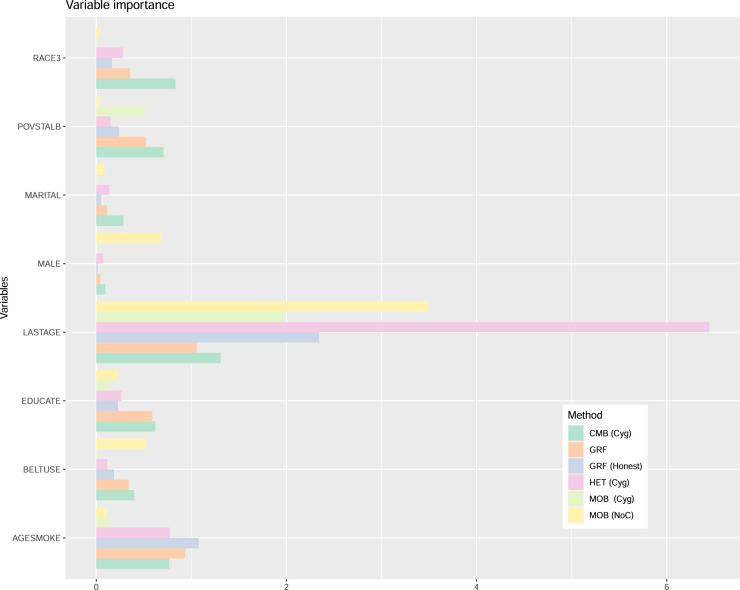
Variable importance for the smoking effect example.

**Figure 6.**
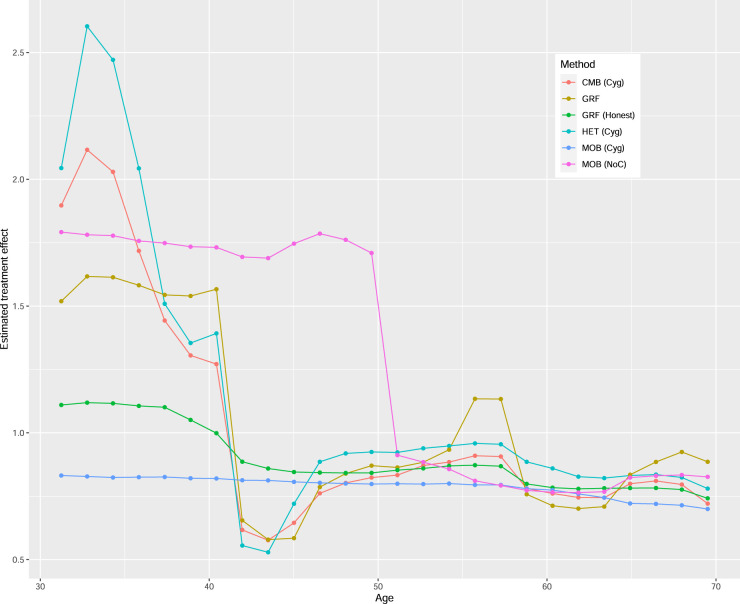
Partial dependence plots of the age (LASTAGE) effect in the example.

Figure 5 presents the variable importance measures. For all methods, LASTAGE is the most important variable. To explore the effect of this variable, Figure 6 displays the partial dependence plots for all methods, focusing on the age range between 30 and 70 years old. The curve for HET exhibits greater variability at lower age values, which corresponds to a region with less available data, making visual analysis more challenging. The complete curves spanning the entire age range are provided in the supplemental material. The general pattern holds true for HET (Cyg), CMB (Cyg), and GRF: the age effect is more pronounced before 40 years old, after which it decreases and stabilizes. However, the pattern diverges for MOB (Cyg), where we observe a small yet consistent (possibly slightly decreasing) treatment effect across all ages. To explore further, we also present the curve for the NoC variant MOB (NoC). Interestingly, this pattern aligns more closely with that of HET (Cyg), CMB (Cyg), and GRF. The supplemental material presents similar graphs for HET (NoC) and CMB (NoC) where we find that the patterns remain the same as for their Cyg variants. Thus, whether we center both the treatment and the response does not significantly impact HET and CMB with this dataset, but it does play a crucial role for MOB. Additionally, Figure 6 also shows the GRF variant with honesty = TRUE, denoted as GRH (Honest). For this variant, the pattern lies somewhere between the one for HET (Cyg), CMB (Cyg), MOB (NoC), and GRF and the one for MOB (Cyg).

These general findings align with those reported in section 7 (see Figure 9 in Hahn et al.^29^), where the data were analyzed using a binary treatment (a binary version of packyears). Notably, the study also highlights that the impact of age on the treatment effect can vary depending on the chosen method. In their analysis, the method specifically designed to detect treatment effects (referred to as BCF) indeed detects a more pronounced heterogeneity moderated by age. They also conclude:

From the above we conclude that how a model treats the age variable would seem to have an outsized impact on the way that predictive patterns are decomposed into treatment effect estimates based on this data, as age plausibly has prognostic, propensity and moderating roles simultaneously.

In our own analysis, both CMB and HET reveal similar treatment effect heterogeneity moderated by age, even if the split rule used by CMB is not specifically designed to increase the heterogeneity of the treatment effect, as opposed to the one used by HET, which is intentionally designed to do so.

These results raise additional methodological and practical questions, which we discuss in the conclusion.

## 5. Conclusion

Based on the findings from our simulation study, the most important recommendation is that locally centering both the response and treatment variables should be the default strategy. Second, all approaches considered perform well. That is, building trees with a split rule that seeks to increase the heterogeneity of the CATE and building trees to predict Y as a proxy target variable are viable approaches. In our scenarios, the hybrid MOB method demonstrated superior performance overall but did not outperform all other approaches consistently. While this recommendation and our findings align with the results reported in Dandl et al.,^16^ they need to be confirmed with further work that will address the limits of our study, as discussed next.

While we explored various scenarios and methods in our simulation study, there are still other aspects that warrant investigation in future research. The first aspect is about the DGPs. The main focus of our study was to investigate the impact of confounder and collider variables. While we considered different versions for the treatment effect τ(x) , our DGPs featured the same μ(x) part, which is called the baseline main effect in Nie and Wager^31^ and the prognostic effect in Dandl et al.^16^ Investigating scenarios where the overall and relative contribution of the prognostic and treatment effect parts vary would be worthwhile and the two aforementioned articles have interesting discussions about this aspect. Moreover, we considered a single sample size and the number of covariates was small. Investigating the impact of sample size and the number of covariates, both noise covariates and the ones related to the response would be interesting. The second aspect is about the methods. While we considered 13 methods, we did not fine-tune the hyperparameters of the RFs and it would be interesting to compare the performance of optimized forests. For instance, the value of nsplit, which determines the number of splits considered for each covariate, was left to its default value of 10 for all forests built with randomForestSRC. This improves computational efficiency but may affect performance in certain cases. Fine-tuning the hyperparameters would likely improve the performance of the methods in absolute terms. However, it would be interesting to investigate whether these adjustments affect the relative performance of the methods.

The results from the real data analysis provide valuable insights for potential future work and practical guidelines. In practice, it is advisable to employ multiple methods and their variants when analyzing the same dataset. This approach serves as a sensitivity analysis. Given that a direct and objective performance measure for estimating treatment effects is unavailable, determining the appropriate course of action when faced with divergent results from different methods would be interesting future work. Additionally, future research could investigate the circumstances under which using the honest version of GRF is preferable.

While the semi-parametric model (2) offers flexibility, it assumes a linear model between Y and G for each given X=x . The rationale is that even if exact linearity does not hold, it might serve as a reasonable approximation in many cases. However, this model has its limitations. Firstly, τ(x) might be a poor estimate of the treatment effect if the link between Y and G is strongly nonlinear for this specific x . Secondly, model (2) assumes that the optimal treatment occurs at one of the boundaries of the possible treatment values. This can be restrictive, especially when seeking to find the optimal treatment value (e.g. determining the optimal drug dose). However, it is possible to allow for a more flexible and nonlinear link between Y and G . Consider for example the following model:

(6) $$E[Y|X=x,G=g]=\beta_{0}(x)+\beta_{1}(x)g+\beta_{2}(x)g^{2}$$

The quadratic formulation allows for greater flexibility and facilitates targeted investigations such as finding the optimal treatment value. Within-node models such as (6) are already feasible with MOB-like methods. It would also be possible to design a split rule that seeks to maximize the heterogeneity of the target parameter of interest. Detailed investigations of these extensions would be interesting for future work. Another possibility is to avoid a parametric assumption altogether and estimate the dose-response function directly. Tree-based methods proposed in Wan et al.^7^ and Nandy et al.^6^ offer such possibilities.

## Supplemental Material

sj-pdf-1-smm-10.1177_09622802241275401 - Supplemental material for Comparison of random forest methods for conditional average treatment effect estimation with a continuous treatment Supplemental material, sj-pdf-1-smm-10.1177_09622802241275401 for Comparison of random forest methods for conditional average treatment effect estimation with a continuous treatment by Sami Tabib and Denis Larocque in Statistical Methods in Medical Research
