# Supplementary material for: Comparison of random forest methods for conditional average treatment effect estimation with a continuous treatment
Source: Stat Methods Med Res. 2024 Oct 9;33(11-12):1952–66. doi: 10.1177/09622802241275401 (PMC11577706; doi:10.1177/09622802241275401)
Supplement: sj-pdf-1-smm-10.1177_09622802241275401 - Supplemental material for Comparison of random forest methods for conditional average treatment effect estimation with a continuous treatment [file sj-pdf-1-smm-10.1177_09622802241275401.pdf]

Supplementary Material for Comparison of Random Forest Methods for Conditional Average Treatment Effect Estimation with a Continuous Treatment

Sami Tabib*, Denis Larocque†
Department of Decision Sciences, HEC Montréal,Canada

*Department of Decision Sciences, HEC Montréal, 3000 chemin de la Côte–Sainte–Catherine, Montréal (Québec), Canada, H3T 2A7. E-mail:sami.tabib@hec.ca

†Corresponding author. Department of Decision Sciences, HEC Montréal, 3000 chemin de la Côte–Sainte–Catherine, Montréal (Québec), Canada, H3T 2A7. E-mail:denis.larocque@hec.ca

1 Monte-Carlo Error and Additional Figures for the Simulation Results Presented in the Article

The simulation study is based on 100 repetitions. This is mainly because the MOB method is very computer intensive. We limited the number of repetitions because we wanted to compare all methods on the same data sets. While 100 repetitions is sufficient to provide the presented conclusions, we ran the simulations for GRF and the best variant (Cyg) for HET and CMB with 1000 repetitions. The results are presented in Figures 1 to 3. We can see that the results are very similar. We also computed the Monte-Carlo error when we use the mean as the summary of the simulation, with the `R` package `Monte.Carlo.se` (Boos et al., 2023). Let $\hat{\theta}$ be the mean of the estimated quantity of interest (here a MSE) over the simulation runs. Let $SE(\hat{\theta})$ be the estimated standard error (i.e. Monte-Carlo error) of $\hat{\theta}$. We define the relative Monte-Carlo error as $SE(\hat{\theta})/\hat{\theta}$. In all scenarios considered in the simulation study, the relative Monte-Carlo error varies between 0.07% and 8.1% with an average of 2.7%, which is relatively small. These facts, along with the fact that several pairwise comparisons tests are significant (see Table 3 in the article) show that 100 repetitions are sufficient to get meaningful conclusions in our study.

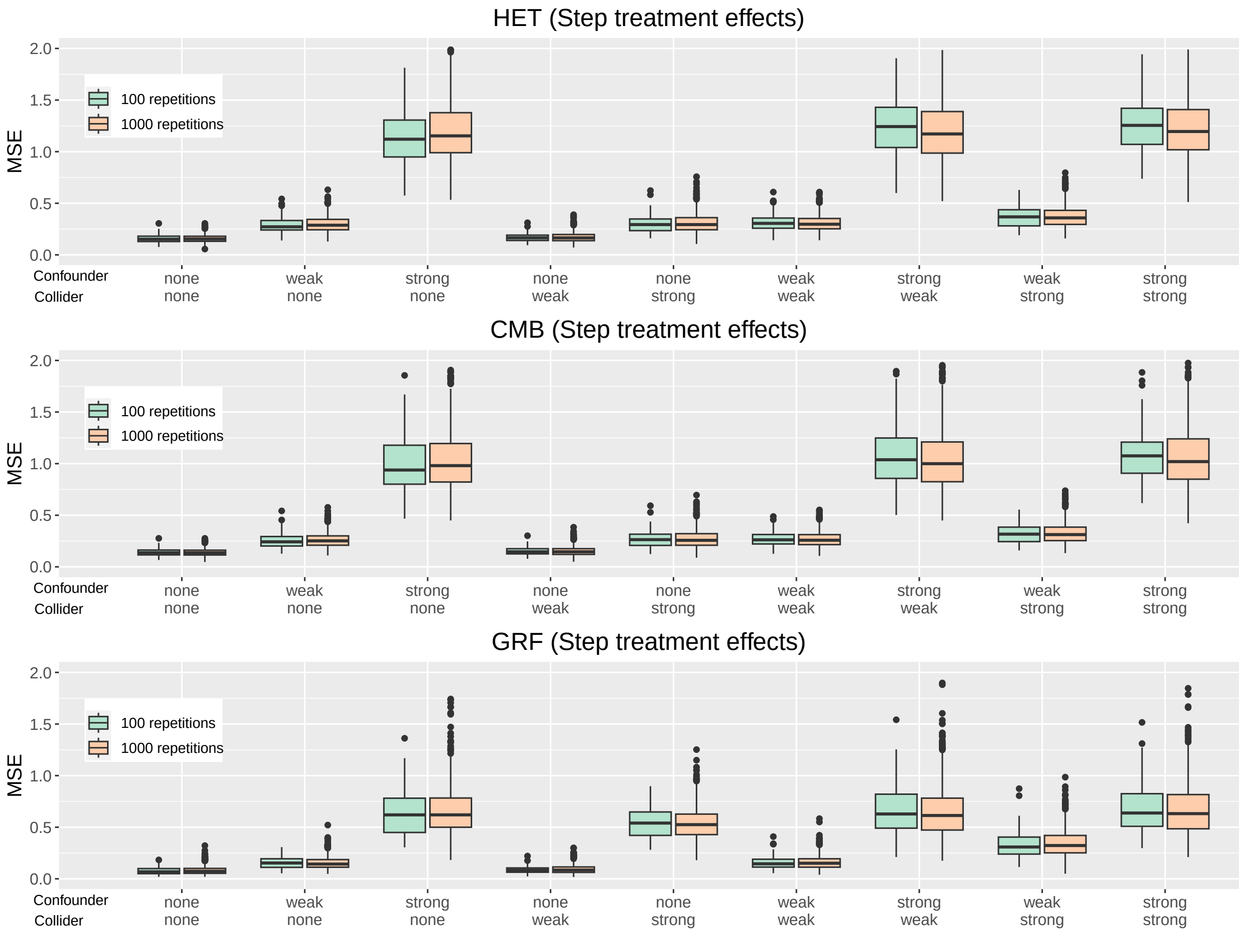

Figure 1: Comparing the results with 100 and 1000 simulation repetitions for the step treatment effects (the Cyg variant are used for HET and CMB)

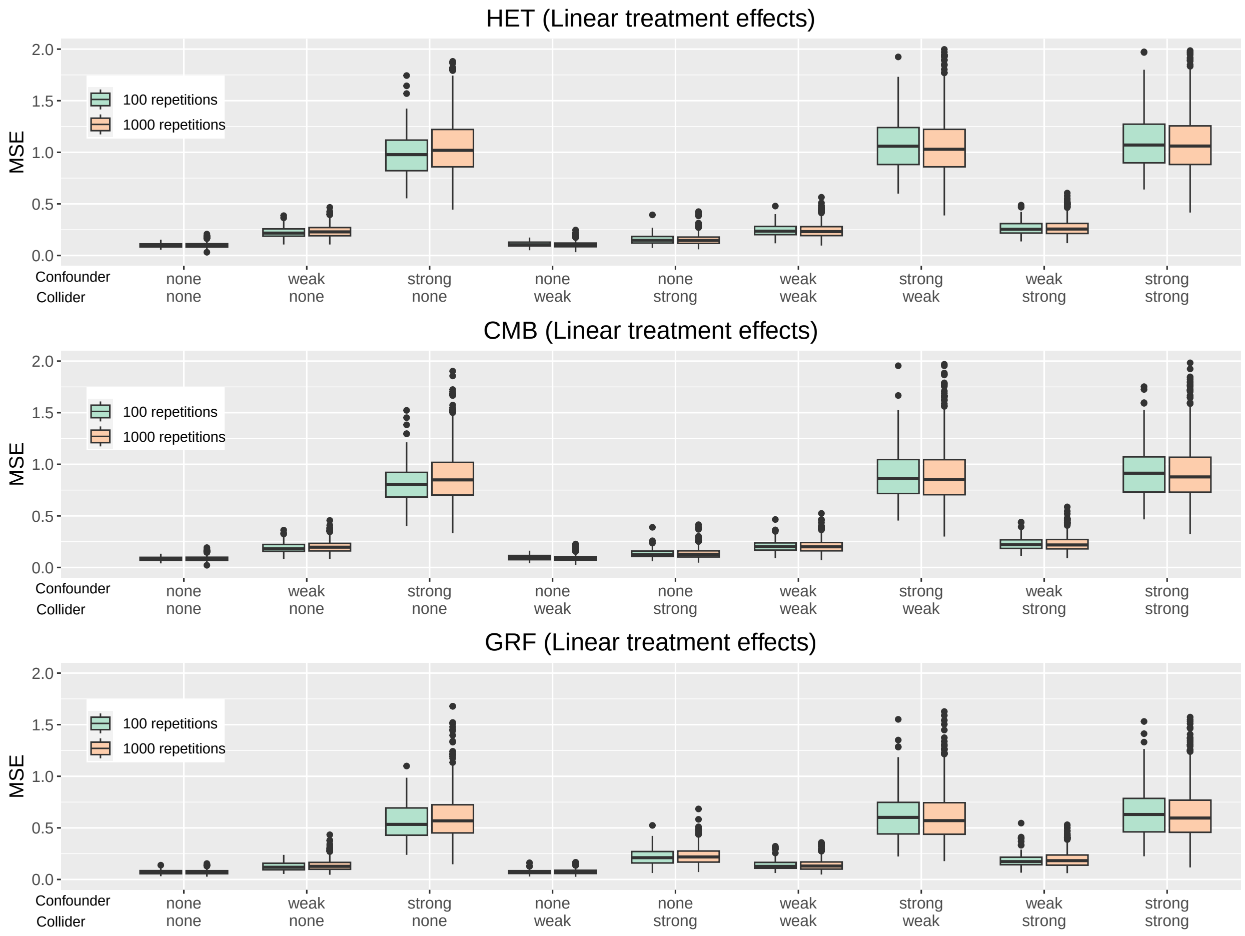

Figure 2: Comparing the results with 100 and 1000 simulation repetitions for the linear treatment effects (the Cyg variant are used for HET and CMB)

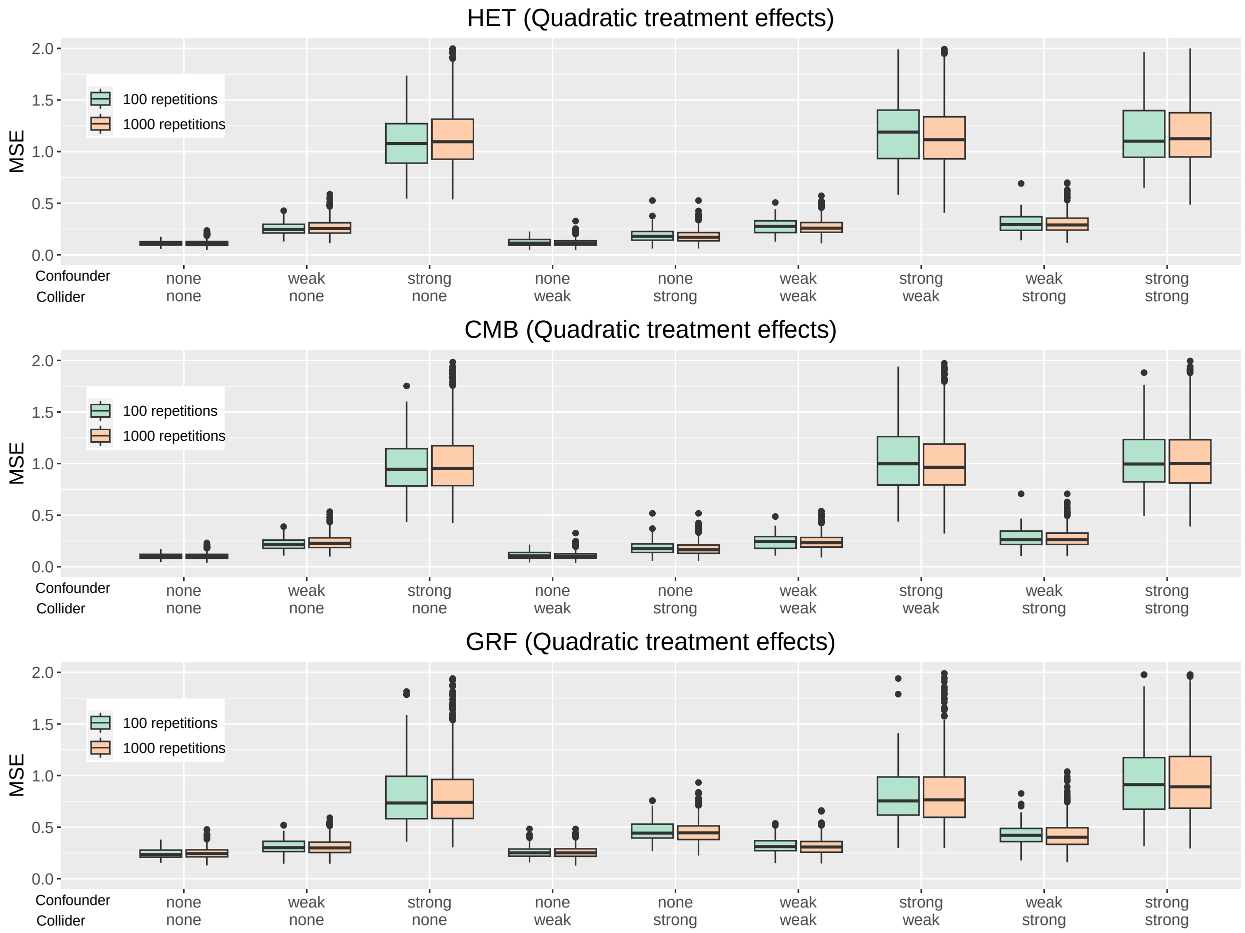

Figure 3: Comparing the results with 100 and 1000 simulation repetitions for the quadratic treatment effects (the Cyg variant are used for HET and CMB)

Figures 1 to 3 in the main article show the performance of all centering variants for a specific method (HET, CMB, and MOB). But to aid interpretation, the y-axis are truncated at 2. The same box-plots, but with an untruncated y-axis are presented in Figures 4 to 6 here.

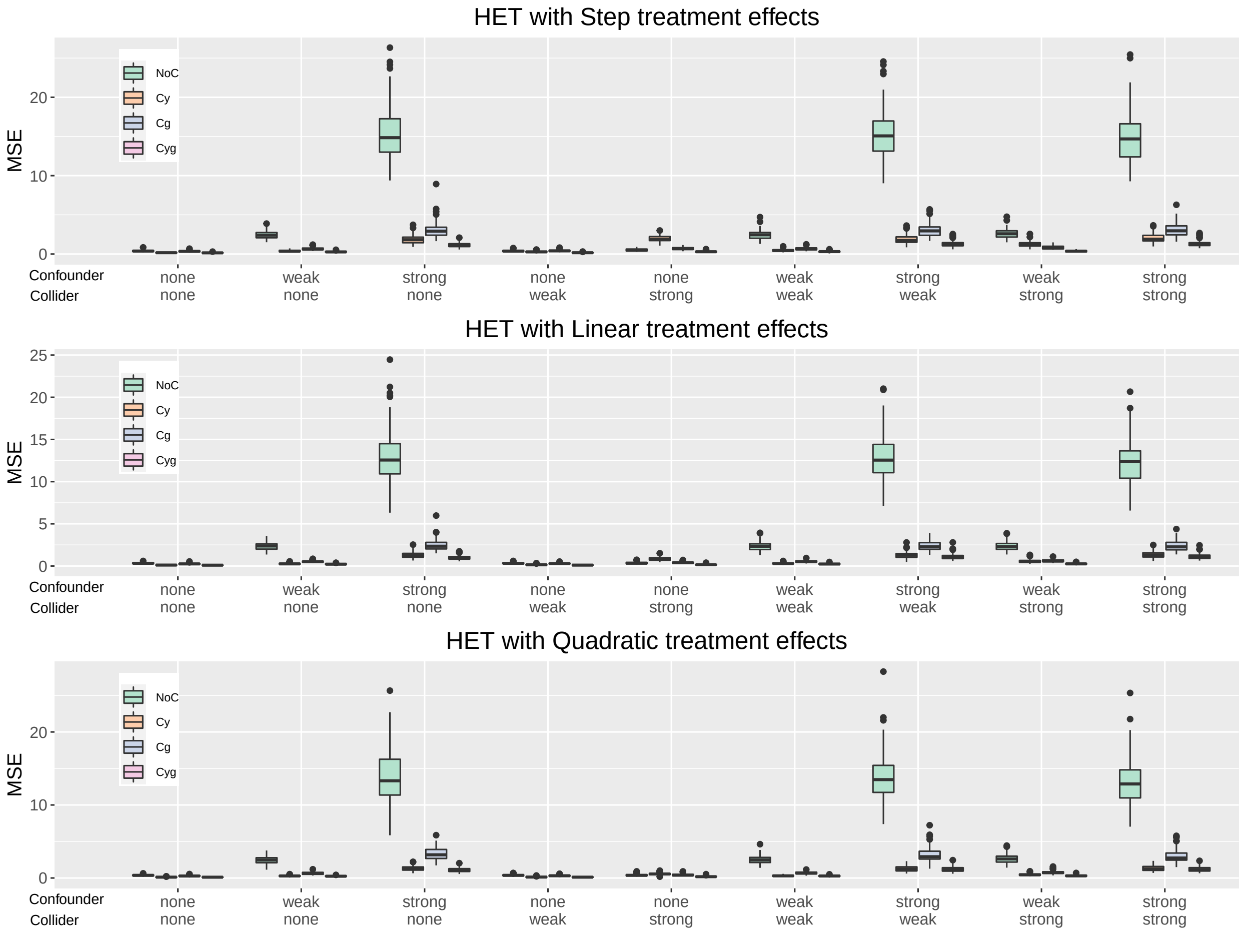

Figure 4: Results for the method HET (untruncated y-axis)

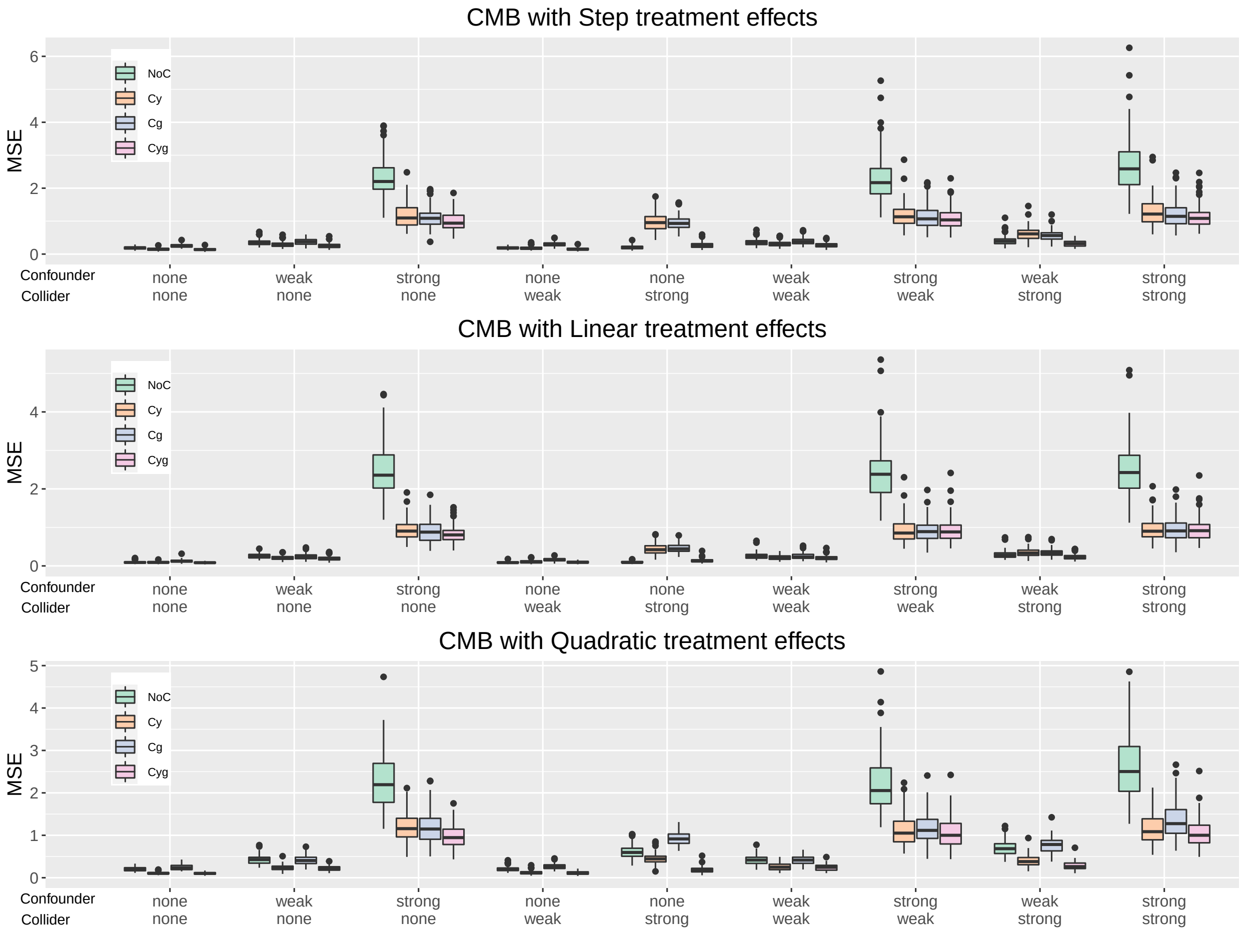

Figure 5: Results for the method CMB (untruncated y-axis)

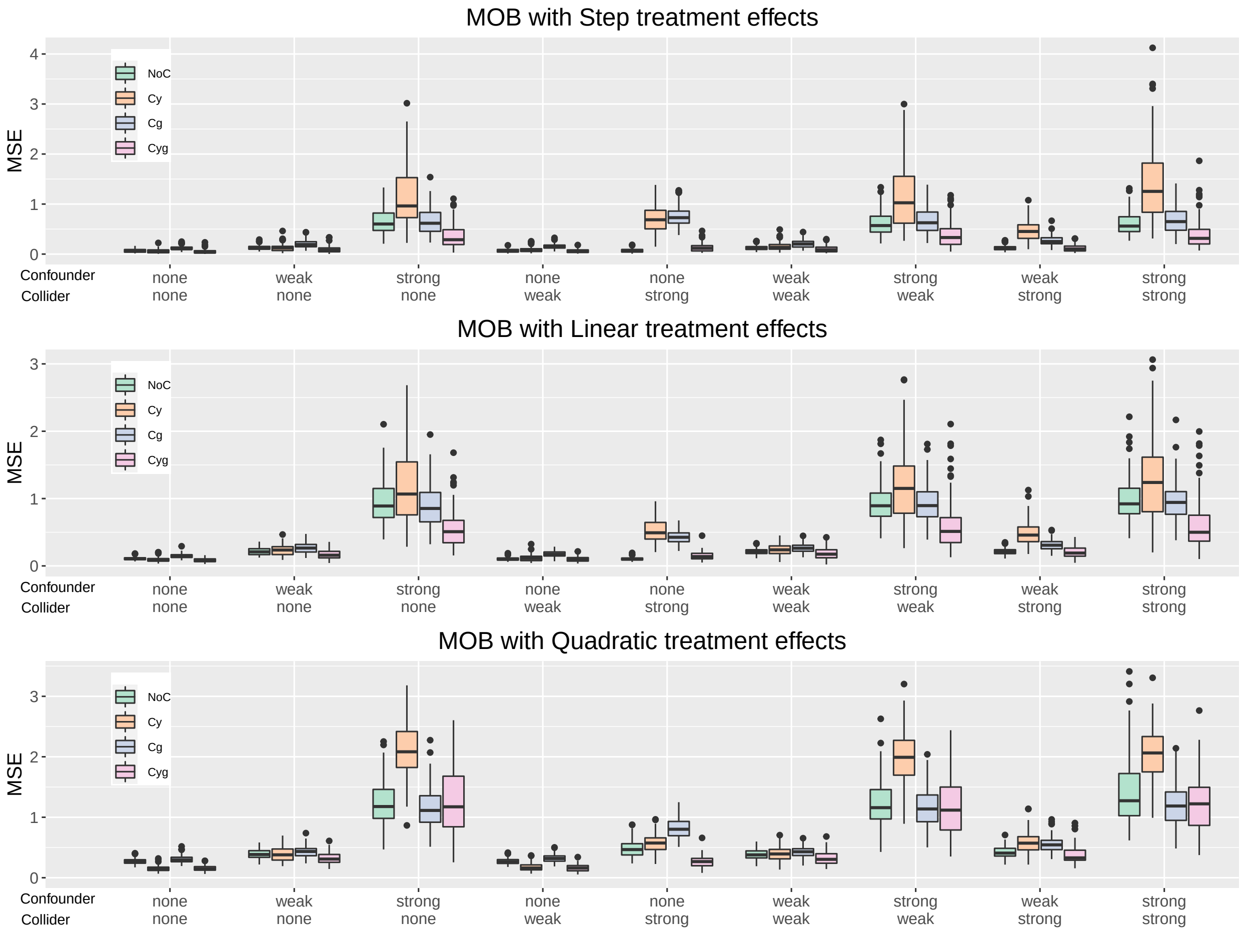

Figure 6: Results for the method MOB (untruncated y-axis)

2 Formal Comparisons with Paired-Sample t-tests Between the Cyg Variant and the Other Variants

For the methods (HET, CMB, and MOB) where different centering variants are investigated, we present the results of paired-sample t-tests, comparing the MSE of the Cyg variant to that of each other variant. These results are summarized in tables 1 to 3. Notably, Cyg consistently outperforms the other variants. Specifically, in 221 out of 243 comparisons, the mean MSE of Cyg is significantly lower. Only in 5 cases does another variant exhibits a significantly lower mean MSE than Cyg.

Method HET	
Cyg better than NoC	27
NoC better than Cyg	0
Difference not significant	0
Cyg better than Cy	26
Cy better than Cyg	0
Difference not significant	1
Cyg better than Cg	27
Cg better than Cyg	0
Difference not significant	0

Table 1: Results of paired-sample t-tests to compare the mean MSE across 100 repetitions for the HET method. Each test was performed at the 5% level. The table reports the number of scenarios in which one method significantly outperforms the other among the 27 scenarios.

Method CMB	
Cyg better than NoC	24
NoC better than Cyg	2
Difference not significant	1
Cyg better than Cy	24
Cy better than Cyg	0
Difference not significant	3
Cyg better than Cg	23
Cg better than Cyg	0
Difference not significant	4

Table 2: Results of paired-sample t-tests to compare the mean MSE across 100 repetitions for the CMB method. Each test was performed at the 5% level. The table reports the number of scenarios in which one method significantly outperforms the other among the 27 scenarios.

Method MOB	
Cyg better than NoC	20
NoC better than Cyg	2
Difference not significant	5
Cyg better than Cy	26
Cy better than Cyg	0
Difference not significant	1
Cyg better than Cg	24
Cg better than Cyg	1
Difference not significant	2

Table 3: Results of paired-sample t-tests to compare the mean MSE across 100 repetitions for the MOB method. Each test was performed at the 5% level. The table reports the number of scenarios in which one method significantly outperforms the other among the 27 scenarios.

3 Comparing the version with `honesty = TRUE` and the one with `honesty = FALSE` for GRF

To provide a fair comparison with the other methods, the main article reports the simulation results for the version with `honesty = FALSE` of GRF. Figure 7 compares directly both versions and shows that the version with `honesty = FALSE` generally performs better. This is mostly the case for the quadratic treatment effects. But there are a few cases where the version with `honesty = TRUE` performs slightly better, especially for the step treatment effects.

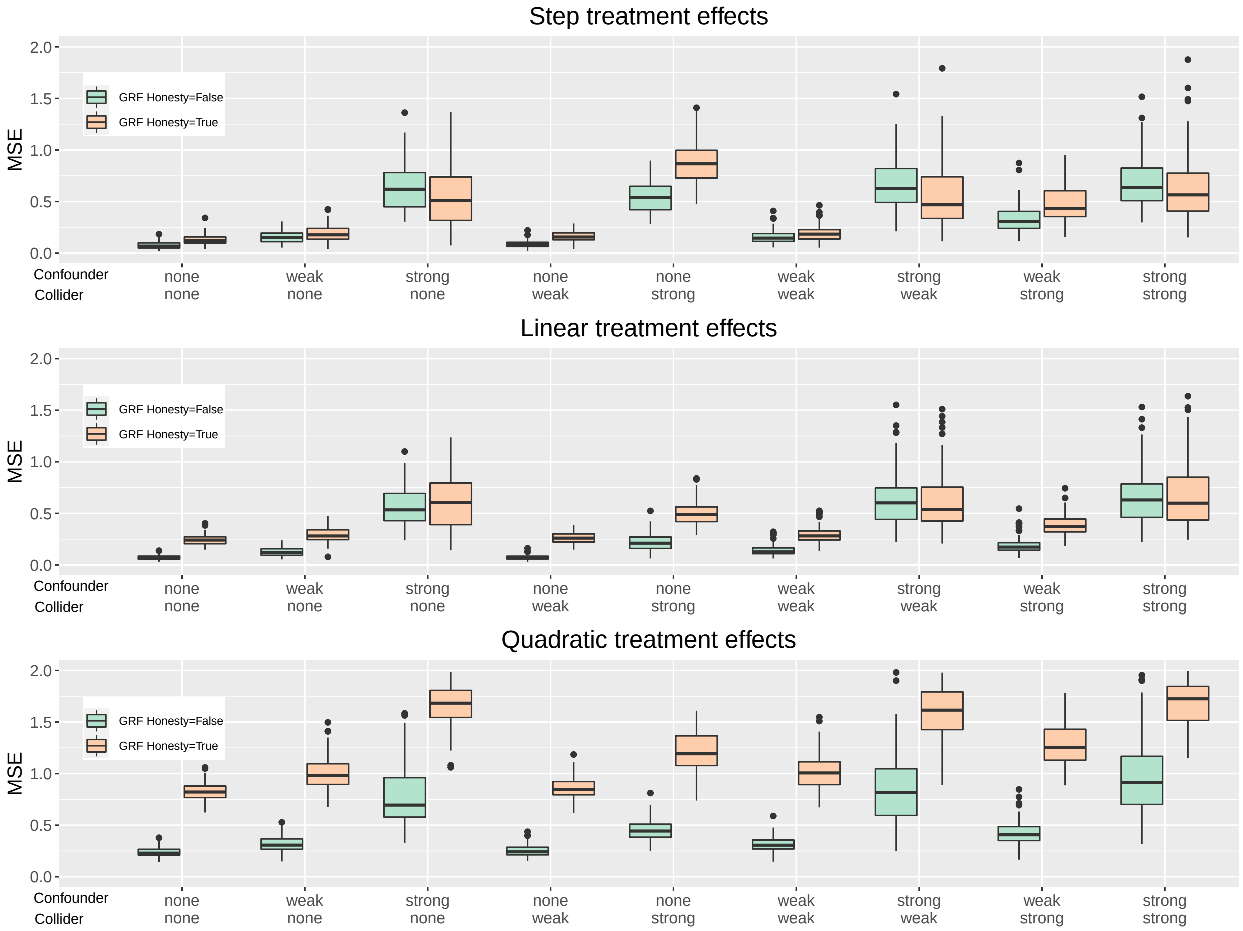

Figure 7: Results for both versions of GRF

4 Simulation Results with Alternative Performance Measures

The main article uses the MSE as the performance measure to compare the methods in the simulation study. Here we present the results with two alternatives metrics, the MAE and the C-index. The MAE (Mean Absolute Error) is given by

$$\text{MAE}_{\text{CATE}} = \frac{1}{n_{test}} \sum_{i=1}^{n_{test}} |\hat{\tau}_i - \tau_i|,$$

where $\hat{\tau}_i$ and τ_i are the estimated and true treatment effects. Smaller values of MSE_{CATE} indicate a better performance.

The C-Index (concordance index) is a ranking metric. In our case, it represents the proportion of observation pairs in the test set where the true and estimated treatment effects are ranked in the same order. Specifically, this applies to pairs that can be compared (i.e. those with $\tau_i \neq \tau_j$). Denoting the indicator function by I, the C-Index can be written as

$$\frac{1}{m} \sum_{i=1}^{n_{test}-1} \sum_{j=i+1}^{n_{test}} \left(I(\tau_i > \tau_j) I(\hat{\tau}_i > \hat{\tau}_j) + I(\tau_i < \tau_j) I(\hat{\tau}_i < \hat{\tau}_j) + I(\tau_i \neq \tau_j) I(\hat{\tau}_i = \hat{\tau}_j)/2 \right),$$

where

$$m = \sum_{i=1}^{n_{test}-1} \sum_{j=i+1}^{n_{test}} (I(\tau_i > \tau_j) + I(\tau_i < \tau_j)).$$

Higher values of the C-index indicate better models. It is important to recognize that the MSE and MAE, on one hand, and the C-Index, on the other hand, measure different characteristics. The MSE and MAE evaluate how close the estimated treatment effects are to the true ones. In contrast, the C-Index evaluates the model's ability to rank observations effectively.

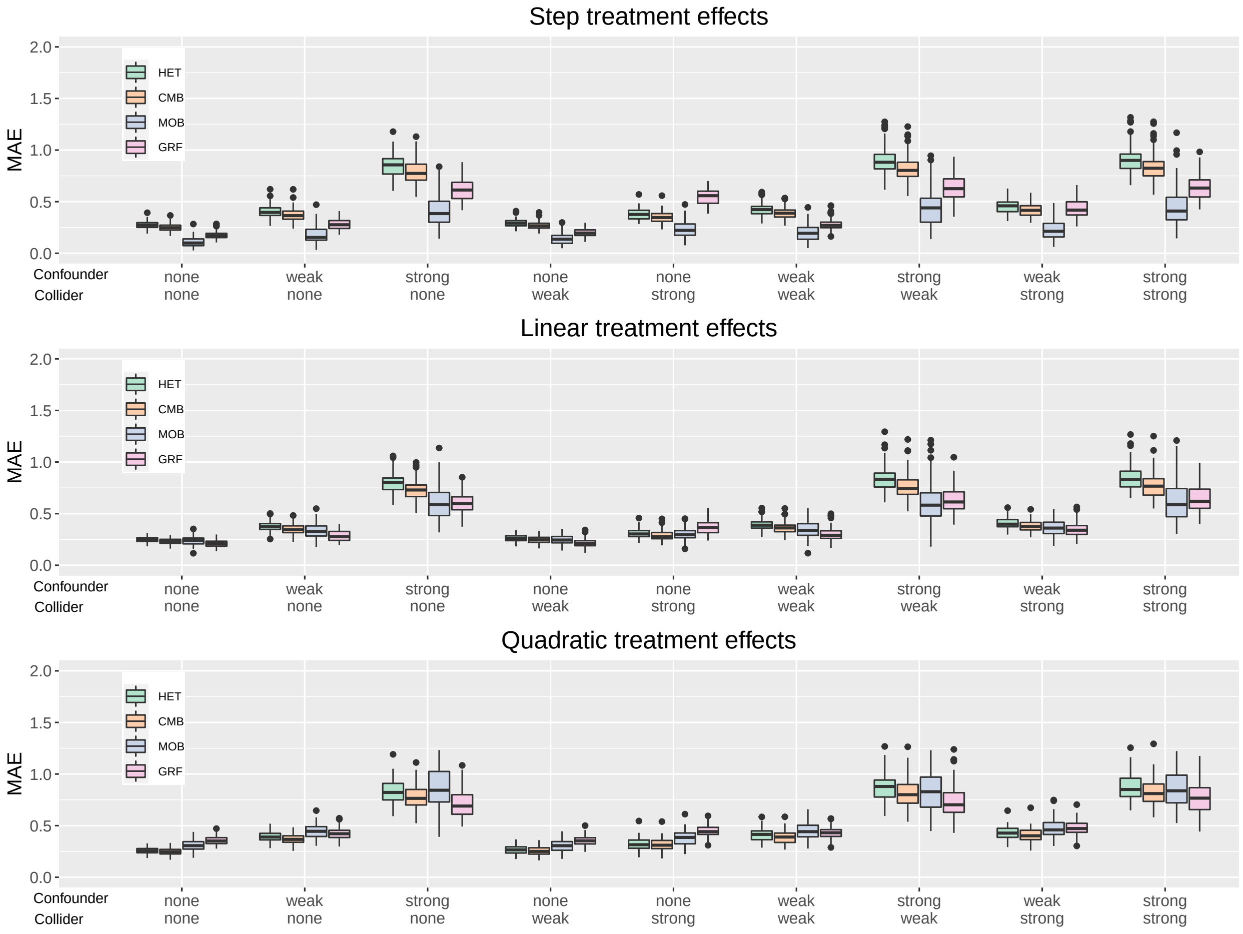

Figure 8: MAE results for all methods, Cyg variant for HET, CMB and MOB

Figure 8 corresponds to Figure 4 in the main article, but it uses the MAE instead of the MSE. The relative rankings of the methods remain the same with the MAE and the same conclusions can be drawn.

Similarly, Figure 9 corresponds to Figure 4 in the main article, but it uses the C-index instead of the MSE. In the case of step treatment effects (upper plot), all methods perform exceptionally well. This outcome is not surprising because there are only two possible treatment effects (1 and 5), which are relatively far apart. The high C-index values indicate that the methods effectively separate observations with a treatment effect of 1 from those with a treatment effect of 5, a relatively straightforward task. This is why measures like

the MAE and MSE are often more discriminative when comparing competing methods. For the linear and quadratic cases, where treatment effects vary continuously, MOB emerges as the best method. Recall (see Table 3 in the main article) that MOB generally outperforms other methods based on the MSE. However, some other methods may achieve substantially lower MSE values than MOB. Interestingly, manual inspection of individual runs revealed instances where MOB had a worse MSE but a better C-Index compared to other methods. This suggests that, in these cases, MOB's ranking ability may be superior, but its estimated treatment effect could be more biased. This is a good illustration that the MSE and C-index capture different characteristics. The C-index results provide new insights but do not change the conclusions reported in the main article.

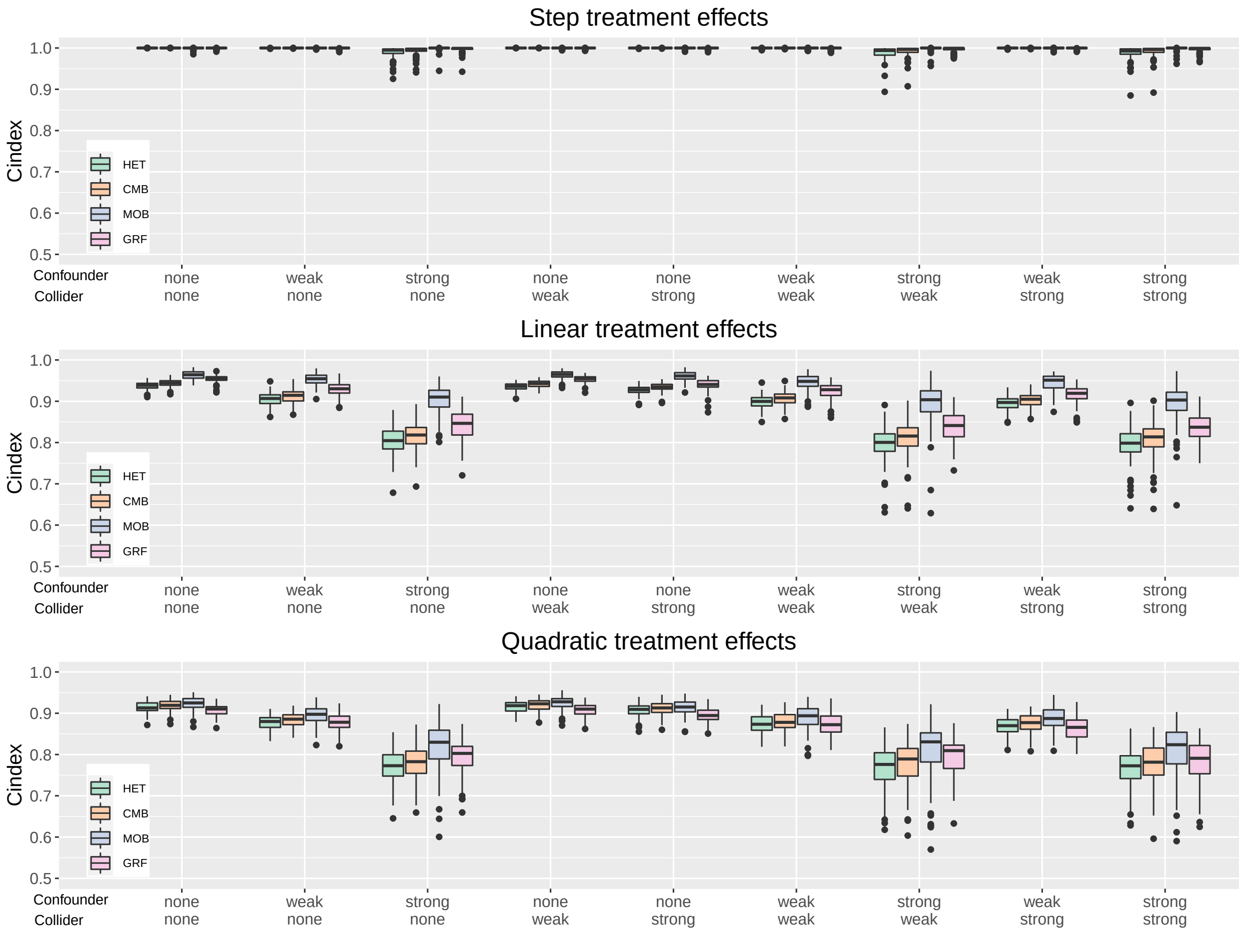

Figure 9: C-index results for all methods, Cyg variant for HET, CMB and MOB

5 Simulation Results with Correlated Covariates

In the simulation study, $X_1, \ldots, X_4$ are independent. The predictive performance of random forests is generally fairly robust to the presence of correlation in the covariates. We ran a simulation for the linear treatment effect by adding correlation to the covariates as a check for the settings considered in our study. In the correlated setting, the pairwise correlations of the covariates $X_1, \ldots, X_4$ is close to 0.6. Figure 10 presents the results for the methods HET, CMB and GRF. Note that MOB was not included in the simulation since it is very computer intensive. The results for the uncorrelated case (the setting in the main article) and the correlated case are very close. This shows that, at least in the settings we considered, correlation does not seem to have an important impact for the predictive performance. In fact, the impact of correlation is usually strong for the variable importance measures (i.e. VIMPs)[1], but this is not the topic of the article.

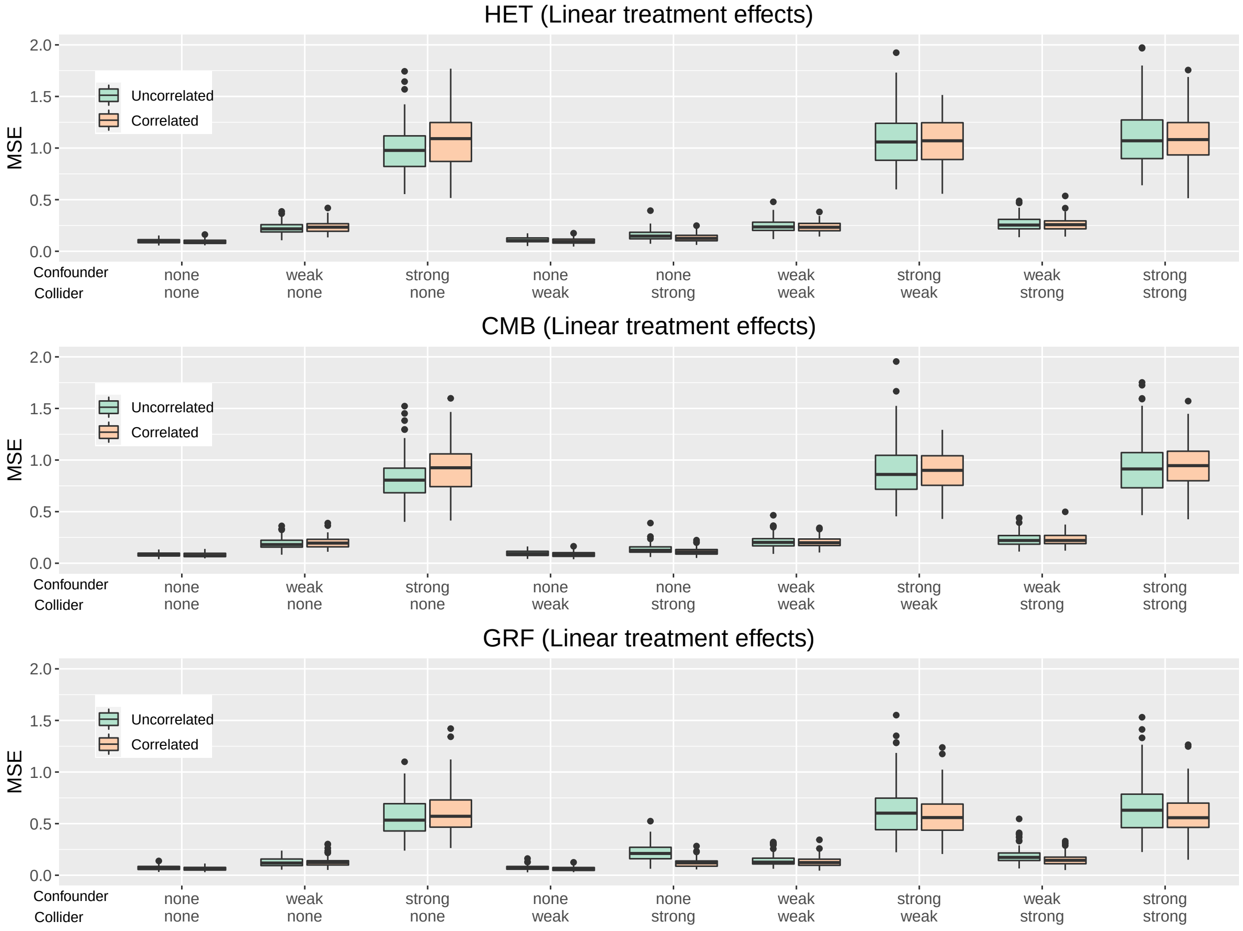

Figure 10: Comparisons between the case where $X_1, \ldots, X_4$ are independent and the case where their pairwise correlations are close to 0.6

[1]Boulesteix, A. L., Janitza, S., Kruppa, J., and König, I. R. (2012). Overview of random forest methodology and practical guidance with emphasis on computational biology and bioinformatics. Wiley Interdisciplinary Reviews: Data Mining and Knowledge Discovery, 2(6), 493-507.

6 Additional Figures for the Real Data Analysis

Figure 6 in the main article displays the partial dependence plots for the variable age (LASTAGE), focusing on the age range between 30 and 70 years old. This is because the curve for HET exhibits greater variability at lower age values, which corresponds to a region with less available data, making visual analysis more difficult. Figure 11 presented here shows the complete curves spanning the entire age range.

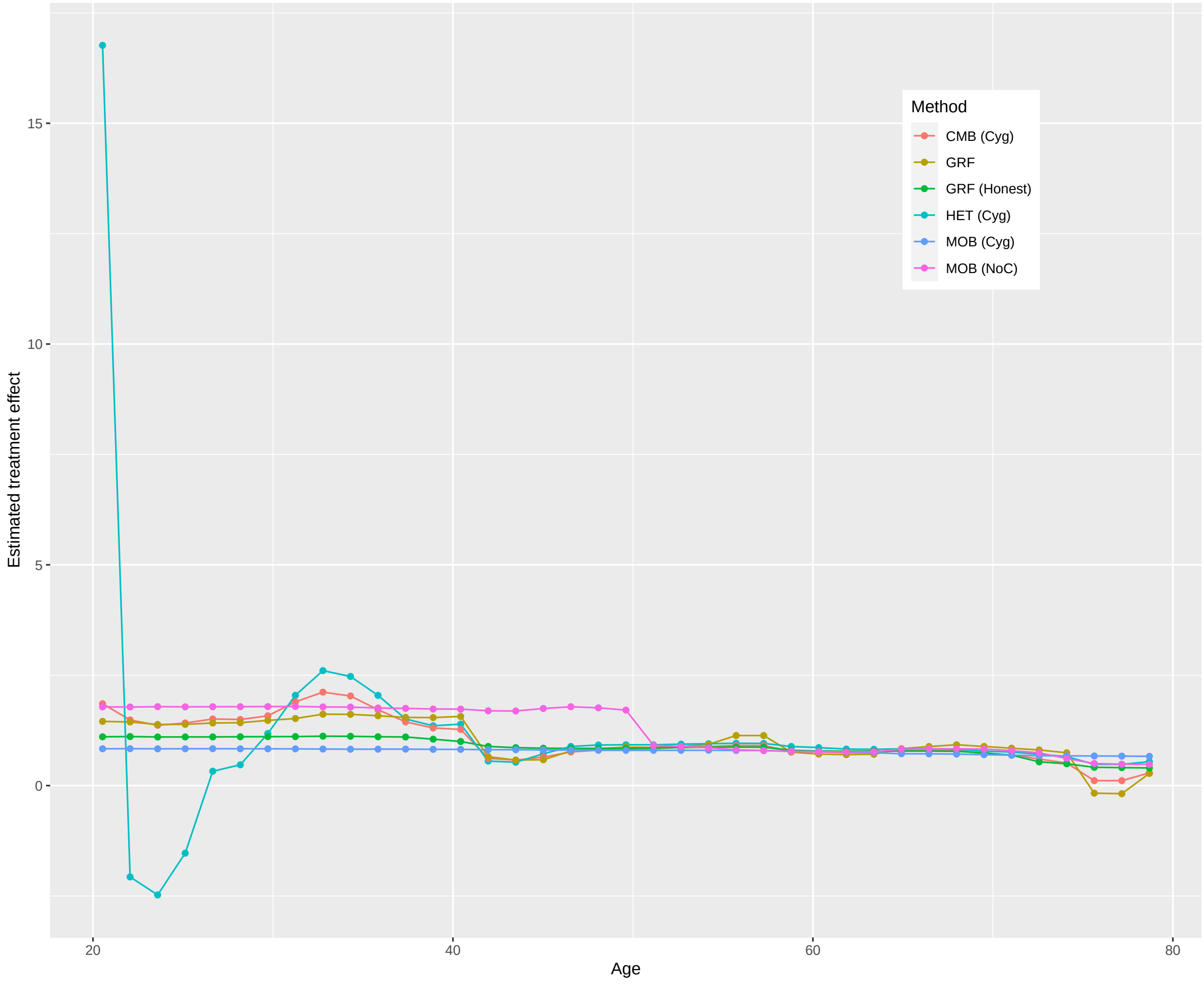

Figure 11: Partial dependence plots of the age (LASTAGE) effect in the example for the whole range of age

In the main article, we observed distinct partial dependence plots for the variable age between the NoC and Cyg variants of the MOB method. However, in Figure 12 presented here, we display the partial dependence plot for the same variable age across the NoC and Cyg variants of the HET and CMB methods. Interestingly, both variants exhibit similar patterns in these methods.

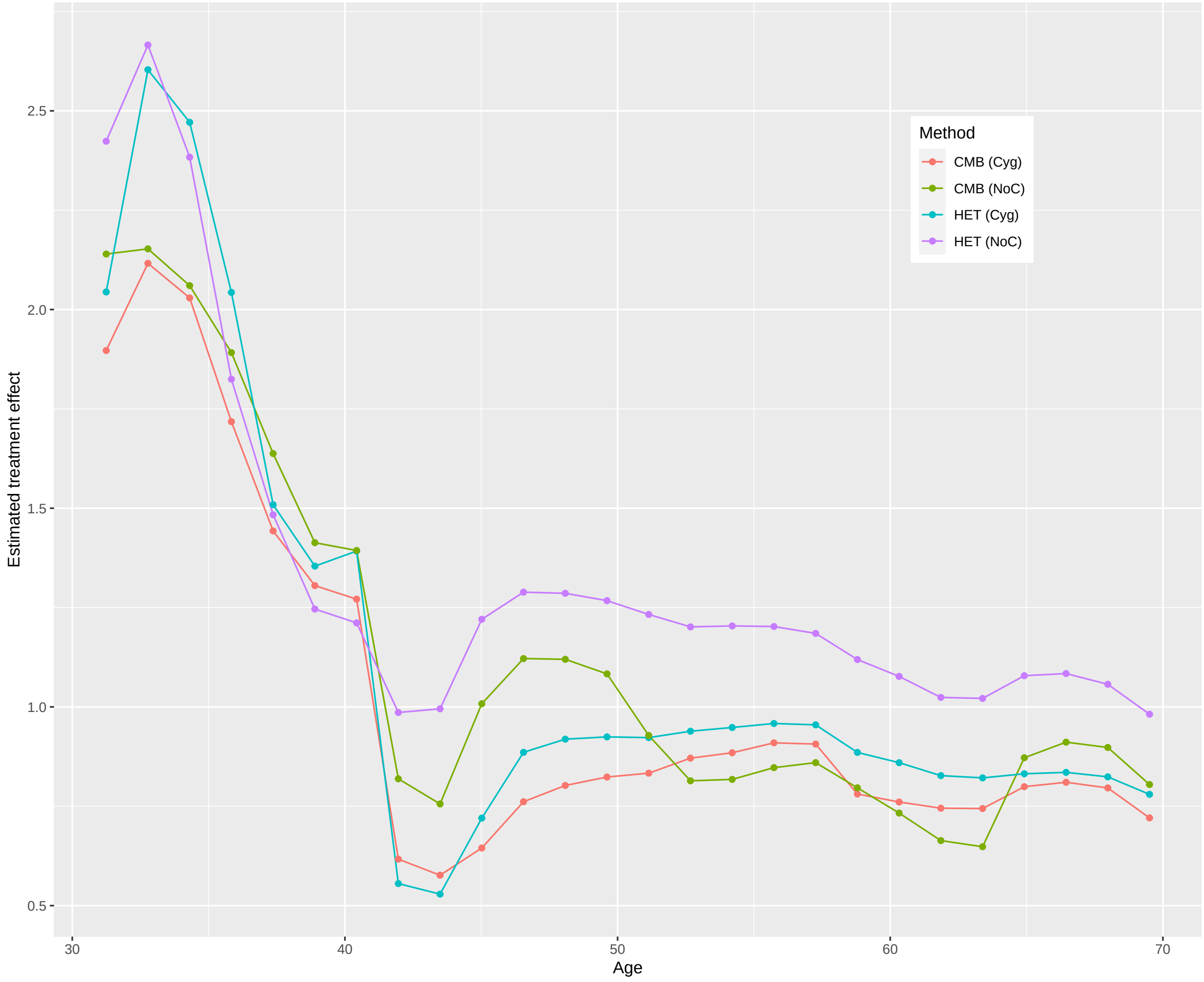

Figure 12: Partial dependence plots of the age (LASTAGE) effect in the example, comparing the locally centered or not variants for CMB and HET

References

Dennis Boos, Kevin Matthew, and Jason Osborne. *Monte.Carlo.se: Monte Carlo Standard Errors*, 2023. URL `https://CRAN.R-project.org/package=Monte.Carlo.se`. R package version 0.1.1.
